# Nivolumab induces seven-year sustained remission in a patient with advanced PD-L1-positive lung adenocarcinoma: a case report

**DOI:** 10.3389/fonc.2026.1742382

**Published:** 2026-02-25

**Authors:** Hongmei Sheng, Wei Zhang, Qihong Yu, Xing Wang, Haiying Peng

**Affiliations:** Department of Respiratory and Critical Care Medicine, Tianjin Chest Hospital, Tianjin, China

**Keywords:** case report, immunotherapy, long-term survival, nivolumab, non-small cell lung cancer, PD-L1

## Abstract

**Objective:**

To summarize the clinical experience of a patient with advanced non-small cell lung cancer (NSCLC) who achieved long-term survival after treatment with the programmed cell death protein 1 (PD-1) inhibitor nivolumab.

**Methods:**

We retrospectively analyzed the case of a 78-year-old male patient diagnosed in April 2018 with right lung adenocarcinoma (cT4N3M1a, stage IV). The patient was driver-gene negative but had a high PD-L1 tumor proportion score (TPS of 90%). He received five cycles of first-line chemotherapy with pemetrexed and cisplatin (PP regimen), which was followed by sequential maintenance therapy with nivolumab (200 mg, Q3W).

**Results:**

After initial response, the disease progressed following first-line chemotherapy. After switching to nivolumab, radiographic evaluation indicated a partial response (PR), which was subsequently assessed as an ongoing response. As of March 2025, the patient remains in continuous remission, with a progression-free survival (PFS) exceeding 82 months and an overall survival (OS) exceeding 84 months. Treatment-related adverse events were mild, and tolerance was excellent.

**Conclusion:**

For patients with PD-L1-high advanced NSCLC, nivolumab monotherapy can induce deep and durable immune responses, enabling long-term survival with a manageable safety profile, even in elderly patients with multiple comorbidities. This case provides compelling real-world evidence for the remarkable efficacy of immune checkpoint inhibitors.

## 1 Introduction

Non-small cell lung cancer (NSCLC) constitutes the most prevalent pathological type of lung cancer, with most patients diagnosed at an advanced stage and a historically poor prognosis (1). In the era of conventional chemotherapy, the median overall survival for patients with advanced NSCLC was approximately one year, with a five-year survival rate of less than 5% (2). The advent of immune checkpoint inhibitors (ICIs) has fundamentally transformed the therapeutic landscape for advanced NSCLC. Nivolumab, a fully human immunoglobulin G4 monoclonal antibody targeting PD-1, reinvigorates T-cell-mediated anti-tumor immunity by blocking the PD-1/programmed death-ligand 1 (PD-L1) pathway (3). Pivotal clinical trials, including CheckMate 017 and 057, established its survival benefit over docetaxel in previously treated patients and demonstrated a characteristic “tail effect” — where responses, once established, can be exceptionally durable, offering a subset of patients the potential for long-term survival or even functional cure (4, 5). Recent reviews have further elaborated on the mechanisms and clinical applications of immune checkpoint inhibitors and their combination strategies (6). Here, we report the case of an elderly patient with PD-L1-high advanced lung adenocarcinoma who has achieved ongoing survival for over seven years following treatment with nivolumab, aiming to offer valuable insights for clinical practice.

## 2 Case presentation

### 2.1 Patient information and history

A 78-year-old male with a smoking history of approximately 40 pack-years presented on April 25, 2018, with a two-month history of intermittent irritating cough, which had recently worsened over ten days and was accompanied by dysphagia, choking, and intermittent dull pain in the right scapular area. Self-administered traditional Chinese medicine and cephalosporin antibiotics provided no relief. A chest CT scan from an external facility revealed a mass near the right hilum and multiple bilateral nodules, suggestive of lung cancer with extensive intra-pulmonary and lymph node metastases. The patient’s medical history included 18 years of diabetes mellitus (controlled with metformin and glipizide), 5 years of hypertension (maximum 150/70 mmHg, controlled with bisoprolol), 18 years of lacunar cerebral infarction without significant sequelae, and 6 years of coronary heart disease status post coronary stent implantation, on long-term aspirin therapy.

### 2.2 Diagnostic assessment

Physical Examination: Vital signs were stable: temperature 36.5 °C, pulse 61 bpm, respiration 18 bpm, blood pressure 126/63 mmHg. The patient was conscious, with no palpable superficial lymphadenopathy. Breath sounds were diminished bilaterally without audible rales. Heart rhythm was regular, abdomen soft and non-tender. No clubbing or pathological reflexes were present.
Laboratory Investigations: Revealed mild anemia (hemoglobin 117 g/L), elevated fibrinogen (4.92 g/L), elevated D-dimer (0.76 μg/mL), and elevated cytokeratin 19 fragment (7.28 ng/mL). Glycated hemoglobin was 6.8%.
Cardiac Ultrasound and Pulmonary Function Tests: Showed aortic sclerosis, segmental left ventricular wall motion abnormality, and low-limit left heart function. Pulmonary function tests indicated normal ventilation, mild diffusion capacity reduction, and moderately increased central airway resistance.
Imaging: A chest contrast-enhanced CT showed a soft-tissue mass in the right upper lobe near the hilum, surrounded by ground-glass opacities, multiple diffuse bilateral nodules of varying sizes, and enlarged mediastinal and bilateral hilar lymph nodes.
Pathology: Bronchoscopy with EBUS-guided biopsy of the right main bronchus lesion and subcarinal lymph node was performed. Pathology confirmed adenocarcinoma in both samples. Genetic testing was negative for EGFR mutations and ALK rearrangements. PD-L1 testing showed high expression (TPS 90%). (Note: Tumor mutational burden [TMB] testing was not performed in this retrospective analysis.).
Final Diagnosis: Stage IV (cT4N3M1a) adenocarcinoma of the right upper lung with bilateral pulmonary metastases and mediastinal/hilar lymph node metastases.

### 2.3 Therapeutic intervention and follow-up

First-line Chemotherapy (May 2018 - August 2018): The patient received five cycles of pemetrexed (0.8g d1) plus cisplatin (40mg d1-2) (PP regimen). Re-evaluation after four cycles showed improvement, but a scan in September 2018 confirmed disease progression (PD).
Second-line Immunotherapy (September 2018 - March 2025): Nivolumab monotherapy (200 mg intravenously every three weeks) was initiated in September 2018. After five cycles (January 2019), a follow-up CT scan showed a partial response (PR), with significant shrinkage of the hilar mass and pulmonary nodules. Treatment was continued (13 cycles in 2019, 3 cycles in 2020, 2 cycles in 2021, and 1 cycle annually from 2022 to 2025). As of the last follow-up in March 2025, the patient remains on therapy. The most recent CT scan shows complete radiographic resolution of the right hilar mass, with only residual streaks and stable nodules, indicating sustained remission.
Safety: During immunotherapy, the patient experienced only Grade 1 fatigue. No Grade 3 or higher immune-related adverse events (irAEs) occurred. Regular monitoring did not reveal biochemical evidence of subclinical irAEs.

**Figure 1:**
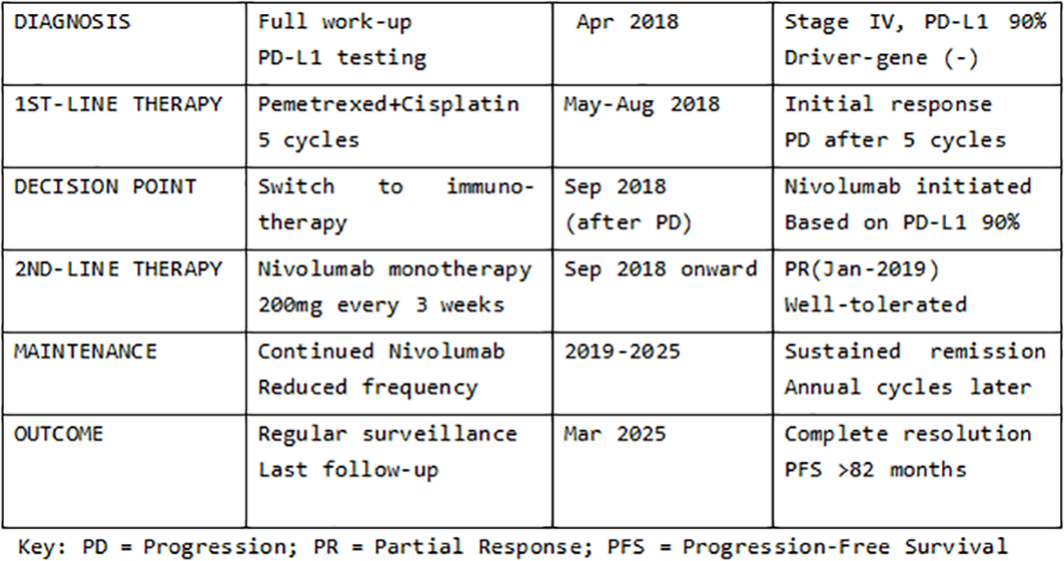
Treatment decision pathway. This flowchart illustrates the complete therapeutic decision-making process from diagnosis to long-term remission, including first-line chemotherapy, treatment switch after disease progression, nivolumab therapy, and long-term maintenance strategy.

**Figure 2:**
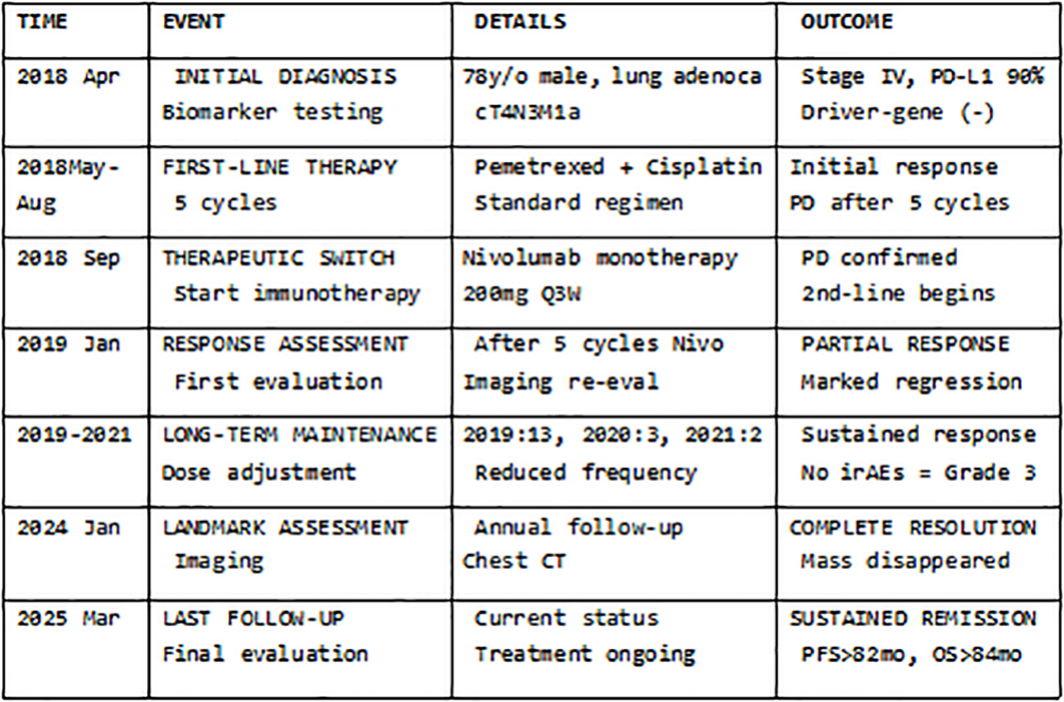
Treatment timeline. This timeline presents the key therapeutic events, imaging assessment time points, and clinical outcomes from 2018 to 2025.

**Figure 3:**
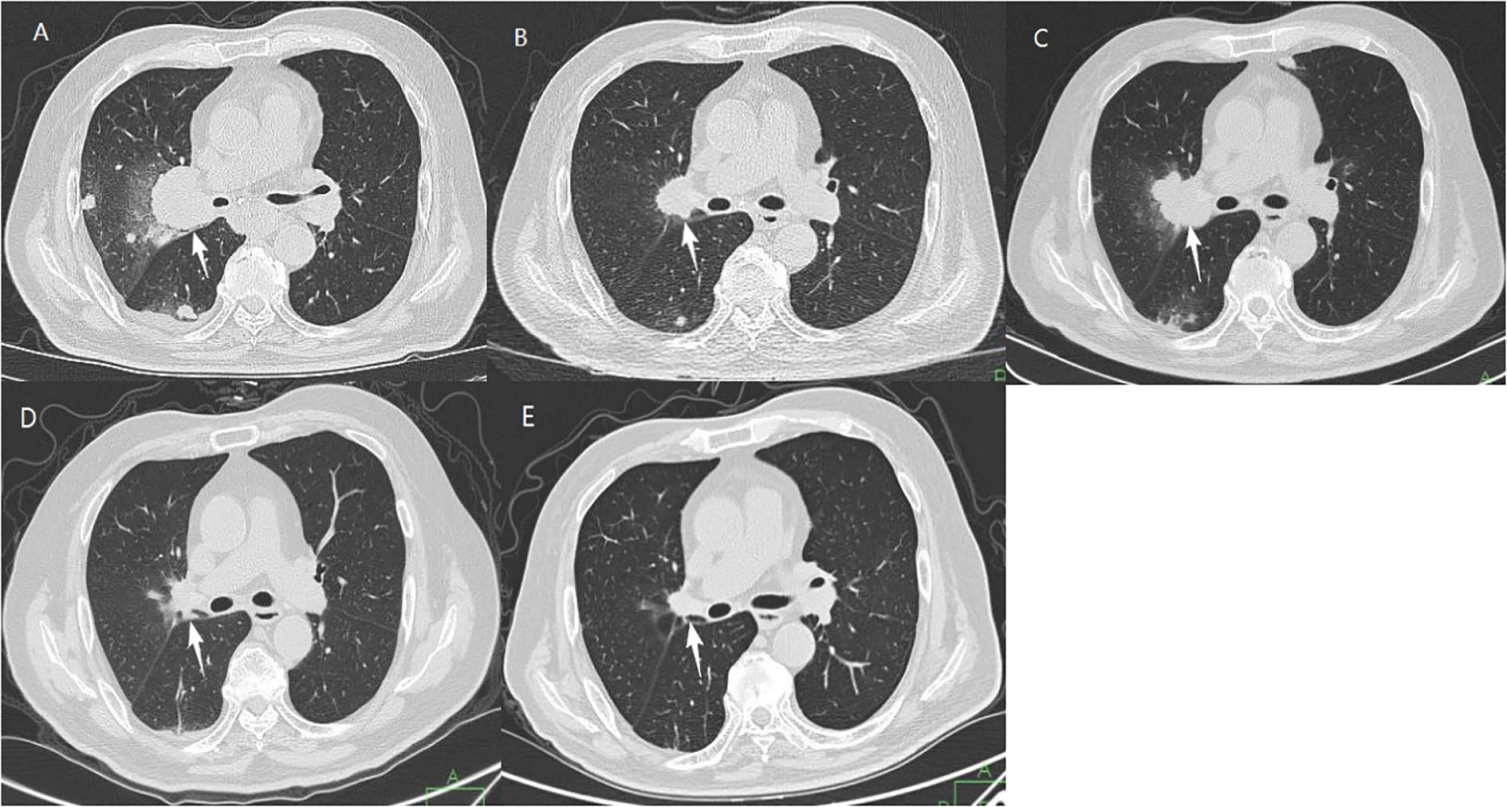
Serial chest CT images illustrating acquired resistance to chemotherapy and a subsequent profound, durable response to nivolumab. **(A)** April 2018, at initial diagnosis, showing a large right hilar mass (white arrow) with multiple bilateral pulmonary nodules. **(B)** July 2018, after 4 cycles of chemotherapy, demonstrating a treatment response with reduction in the size of the lesions. **(C)** September 2018, prior to the 5th cycle, confirming disease progression compared to **(B)**, indicating acquired resistance. **(D)** January 2019, after 5 cycles of nivolumab, showing a partial response with marked regression. **(E)** March 2025, after 7 years of immunotherapy, showing sustained complete radiographic resolution.

The entire diagnostic and therapeutic journey is summarized in **Figures 1** and **2**, with serial CT images shown in **Figure 3**.

## 3 Discussion

This case of an elderly patient with multiple comorbidities and PD-L1-high (TPS 90%) advanced lung adenocarcinoma who achieved over seven years of continuous remission with nivolumab offers several key clinical insights.

### 3.1 Strengths of this case

First, this case provides rare long-term real-world evidence of continuous remission exceeding seven years with nivolumab monotherapy in a patient with exceptionally high PD-L1 expression (TPS 90%), vividly illustrating the characteristic “tail effect” of immunotherapy. Second, the patient was 78 years old with multiple significant comorbidities (coronary heart disease, diabetes, hypertension), yet achieved excellent efficacy and tolerability, challenging conventional hesitations about using immunotherapy in such complex elderly patients and providing valuable management experience (5, 7). Finally, the complete series of CT images provides clear and compelling visual evidence of this dramatic therapeutic response.

### 3.2 Limitations of this study

This study is a retrospective case report with inherent limitations. First, we did not perform tumor mutational burden (TMB) testing, preventing a comprehensive analysis of its association with ultra-long survival. Second, as a retrospective analysis, our assessment of immune-related adverse events (irAEs) was based on medical records rather than prospective systematic scale evaluations. Most importantly, a single case report cannot provide mechanistic evidence for synergistic effects between sequential chemotherapy and immunotherapy. These limitations also point to directions for future prospective research.

### 3.3 Corroboration with previous studies and implications

The high response rate in this case strongly correlates with high PD-L1 expression, reaffirming PD-L1’s value as an important predictive biomarker (5, 8). Interestingly, the patient responded profoundly to subsequent immunotherapy despite progression after first-line chemotherapy. Some basic research suggests that chemotherapy may alter the tumor microenvironment by inducing immunogenic cell death, potentially creating favorable conditions for subsequent immunotherapy (9). However, it must be clearly stated that this case itself cannot provide direct mechanistic evidence supporting this hypothesis; this viewpoint originates from previous literature, and our case serves merely as an illustration of this clinical phenomenon. Future prospective studies are needed to validate the mechanisms and advantages of this sequential strategy.

## 4 Conclusion

This case provides powerful evidence that nivolumab monotherapy can induce profound and durable responses in patients with PD-L1-high advanced NSCLC, leading to exceptional long-term survival. It demonstrates that such benefits are achievable in elderly patients with complex medical histories, with a manageable safety profile. This report offers clinicians a highly valuable reference for the real-world application of immune checkpoint inhibitors.

## Data Availability

The datasets presented in this article are not readily available because they consist of confidential patient medical records. Requests to access the datasets should be directed to the corresponding author and will require approval from the institutional ethics committee and a data sharing agreement. Requests to access these datasets should be directed to Haiying Peng; xuefeihong520@126.com.
